# Red meat consumption and risk for dyslipidaemia and inflammation: A systematic review and meta-analysis

**DOI:** 10.3389/fcvm.2022.996467

**Published:** 2022-09-30

**Authors:** Le Sun, Jia-Lin Yuan, Qiu-Cen Chen, Wen-Kang Xiao, Gui-Ping Ma, Jia-Hua Liang, Xiao-Kun Chen, Song Wang, Xiao-Xiong Zhou, Hui Wu, Chuang-Xiong Hong

**Affiliations:** ^1^Guangzhou University of Chinese Medicine, Guangzhou, China; ^2^The Department of Cardiovascular Disease, Meizhou Hospital of Traditional Chinese Medicine, Meizhou, China; ^3^The Department of Cardiovascular Disease, The First Affiliated Hospital of Guangzhou University of Chinese Medicine, Guangzhou, China

**Keywords:** red meat, lipids, dyslipidaemia, inflammation, meta-analysis

## Abstract

**Aim:**

The study (PROSPERO: CRD42021240905) aims to reveal the relationships among red meat, serum lipids and inflammatory biomarkers.

**Methods and results:**

PubMed, EMBASE and the Cochrane databases were explored through December 2021 to identify 574 studies about red meat and serum lipids markers including total cholesterol (TC), triglyceride (TG), low-density lipoprotein cholesterol (LDL-C), high-density lipoprotein cholesterol (HDL-C), C-reactive protein (CRP) or hypersensitive-CRP (hs-CRP). Finally, 20 randomized controlled trials (RCTs) involving 1001 people were included, red meat and serum lipid markers and their relevant information was extracted. The pooled standard mean difference (SMD) was obtained by applying a random-effects model, and subgroup analyses and meta-regression were employed to explain the heterogeneity. Compared with white meat or grain diets, the gross results showed that the consumption of red meat increased serum lipid concentrations like TG (0.29 mmol/L, 95% CI 0.14, 0.44,*P*<0.001), but did not significantly influence the TC (0.13 mmol/L, 95% CI −0.07, 0.33, *P* = 0.21), LDL-C (0.11 mmol/L, 95% CI −0.23, 0.45, *P* = 0.53), HDL-C (−0.07 mmol/L, 95% CI −0.31, 0.17, *P* = 0.57),CRP or hs-CRP (0.13 mmol/L, 95% CI −0.10, 0.37,*P* = 0.273).

**Conclusion:**

Our study provided evidence to the fact that red meat consumption affected serum lipids levels like TG, but almost had no effect on TC, LDL-C, HDL-C and CRP or hs-CRP. Such diets with red meat should be taken seriously to avoid the problem of high lipid profiles.

**Systematic review registration:**

[https://www.crd.york.ac.uk/PROSPERO], identifier [CRD42021240905].

## Introduction

Red meat includes edible animal muscle from cows, pigs, and sheep, and it is a favorite food for most people worldwide (1, 2). In recent years, some groups have urged people to consume plant-derived foods rather than animal-derived foods (3). Red meat is considered as a kind of high-quality protein with many other beneficial nutrients, such as fatty acids, vitamins, minerals and molecules mediating various cellular responses (1, 4, 5). However, excessive intake of red meat also gives rise to abnormalities in lipid metabolism, inflammatory reactions and possibly chronic diseases (6). Serum total cholesterol levels change if there is excessive consumption of cholesterol and saturated fats, and high levels of serum cholesterol accumulates in macrophages and then activates the NLRP3 inflammasome through the NF-κB signaling pathway (6, 7).

**FIGURE 1:**
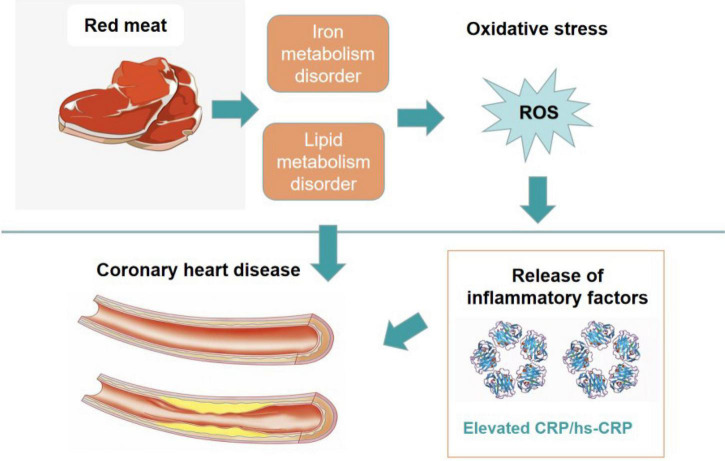
Mechanism of lipid metabolism and inflammatory reaction induced by red meat.

On the other hand, dyslipidaemia is becoming a concern worldwide, and it has been proven to be a major risk factor for cardiovascular and metabolic diseases and the underlying cause of stroke and other life-threatening diseases (8–10). In recent years, chronic inflammation has been proven to be the trigger of abnormal lipid metabolism (11). Oxidative stress triggers inflammation, and a study on the consumption of red meat concluded that red meat could give rise to changes in oxidative stress and further induce inflammation and related diseases (12, 13). In addition, red meat is the major source of serum iron, especially for the meats with high myoglobin content (14). However, excessive intake of iron ions in human body may trigger oxidative stress and aggravate inflammatory reaction (2) (Figure 1).

Lipoproteins in the blood like low-density lipoprotein cholesterol (LDL-C) can enter the arterial intima from the circulation, and the accumulation of lipoproteins in the arterial intima can trigger inflammation and induce pathological changes that threaten people’s lives and health (15–17). In contrast to lipoproteins, oxidized lipids (ox-LDL) are considered to have a much stronger influence on inflammation; ox-LDL can not only be synthesized endogenously but can also be obtained through the diets (18). Therefore, inhibiting proinflammatory cytokines has emerged as a novel promising mode of therapy to improve and complement the current lipid-lowering approaches (7).

Some studies, especially those supporting the US Dietary Guidelines for Americans, demonstrated that daily consumption of red and processed meat might increase the risk of coronary heart disease (CHD) (19). A proposal in emphasized a transformation trend to a daily diet that consisted mainly of plant-derived foods (20). Similarly, a study from Boston conducted a follow-up with 1,023,872 people, comparing the effect of red meat with other dietary components, such as legumes and grain. The results showed that a greater intake of red meat was positively correlated with a relatively higher risk of CHD (21).

However, recent studies hold the opposite view: a large prospective study conducted by The Netherlands Cohort Study (NLCS) found that red meat intake does not increase the risk of cardiovascular and respiratory mortality (22). Another article published in the *Annals of Internal Medicine* found that there is not enough scientific evidence to establish a link between the intake of red meat and cardiometabolic diseases (23).

Therefore, our study aimed to provide relevant evidence about the effects of the consumption of red meat on serum lipid levels and inflammatory markers.

## Materials and methods

This systematic review was registered at the International Prospective Register of Systematic Reviews (PROSPERO) (registration number: CRD42021240905).

### Patient and public involvement statement

We conducted the systematic review and meta-analysis through exploring studies on

databases and there were no additional patients or public involvements needed, all inclusion criteria were consistent with the original study.

#### Search strategy

Literature searches were conducted in three databases: PubMed, EMBASE, and the Cochrane Central Register of Controlled Trials (through 14 December 2021). Two authors (Y.J.L. and X.W.K.) independently searched the databases by using standardized terms without year and language restrictions, including: Group 1) “red meat,” “red meats,” “beef,” “pork,” “lamb”; Group 2) “randomized controlled trial,” “randomized,” “placebo”; Group 3) keywords for lipid-related markers: Adiponectin, Adipocyte Complement-Related Protein 30 kDa, Adipocyte Complement Related Protein 30 kDa, Adipose Most Abundant Gene Transcript 1, apM-1 Protein, apM 1 Protein, ACRP30 Protein, Adipokynes, Adipocyte, Cytokines, IL-1β, IL-6, TNF-α, CRP, c-Reactive protein, Interleukin, Triacylglycerol, Triacylglycerols, Triglyceride, Triglycerides, Dyslipidaemia, Dyslipoproteinemias, Dyslipoproteinemia, Blood lipid, HDL lipoproteins, High density lipoprotein, Lipoprotein, Lipoproteins, High density lipoproteins, Alpha-lipoproteins, Alpha-lipoprotein, Heavy lipoproteins, Alpha-1 lipoprotein, HDL, Low density lipoprotein cholesterol, Low density lipoprotein, Low density lipoproteins, Low-density lipoprotein, Beta-lipoprotein cholesterol, Cholesterol, Beta lipoprotein, Beta-lipoproteins, Beta lipoproteins, Beta lipoprotein cholesterol, LDL lipoproteins, LDL cholesterol, Cholesteryl linoleate, LDL, LDL cholesteryl linoleate, LDL. Each database was searched using keywords in Group 1 combined with the terms in Groups 2 and 3. Then, inappropriate articles were excluded by manual screening.

#### Eligibility criteria

Articles were included if they met the following criteria: (1) Randomized controlled trial (RCT) including parallel or crossover designs; (2) people recruited met the age restriction ≥ 18 years; (3) the intervention in one group was red meat, including beef, pork, lamb and mutton, and the other group was given non-red meat, including chicken, fish, soy, etc.; (4) the outcomes included at least one of the lipid parameters (LDL-C, HDL-C, TC, and TG); (5) mean and standard deviation (SD) were provided. The exclusion criteria were as follows: (1) recruited subjects were children, or the pregnant women; (2) the intervention had other programs which may influence the serum lipids levels, like walking or exercise training, etc.; (3) unclear habitual diet; (4) all participants are postmenopausal women.

#### Data extraction

Our team included 7 investigators guided by H.C.X, and two authors (Y.J.L. and X.W.K.) first conducted the study inclusion process by independently reading the titles and abstracts. If there were any discrepancies, the other authors (S.L. and L.J.H) were consulted. We identified 574 relevant studies on this topic, and all of the included articles had their relative characteristics extracted, including the first author’s name, publication year, country, population size, gender ratio, health condition, mean BMI or body weight, mean age and study design, intervention meat, control alternatives, study duration, and change before and after the intervention of the serum lipids and inflammation index, such as total cholesterol (TC), triglyceride (TG), low-density lipoprotein cholesterol (LDL-C), high-density lipoprotein cholesterol (HDL-C), C-reactive protein (CRP) and hypersensitive-CRP (hs-CRP).

#### Quality assessment

Risk of bias was assessed by two authors (L.J.H. and M.G.P.) with the Cochrane risk-of-bias tool (RoB2), which considers the statistical analyses including the randomization method, allocation scheme concealment, blinding method, outcome data integrity, selective research results, other bias sources and the overall bias.

#### Statistical analyses

For the parallel or crossover trial design studies, we included the preintervention data and the final overall data, including means and standard deviations. For the analysis, all of the studies generally could be considered parallel designs of the respective groups, and if there were more than one intervention group or control group, we tended to adopt the data from the red meat groups and non-red meat alternative groups to analyze the differences between them (24, 25). The pooled standard mean difference (SMD) was obtained by meta-analyses of binary and continuous meta functions with a random-effects model after checking the heterogeneity. In terms of the heterogeneity among the studies, we used the I^2^ and Q statistics (26, 27). For the Q statistics, *P*<0.10 showed significant heterogeneity, and I^2^ values of 25%, 25-50%, 50-70%, and ¿75% were classified as indicating no, small, moderate, and significant heterogeneity, respectively. Moreover, we performed subgroup analysis by using the publication year, country, population size, gender, health condition, mean BMI or body weight, mean age and study design, intervention meat, control alternatives, and study duration to explore any heterogeneity.

We also performed meta-regression to examine the effect of potential factors on the serum TC concentration, and to assess the potential publication bias, we used Egger’s linear regression test. Sensitivity analyses were carried out by excluding each study one by one and re-analyzing the data. All statistical analyses were performed with STATA 13.0 (Stata Corp.).

## Results

### Literature searches

**FIGURE 2:**
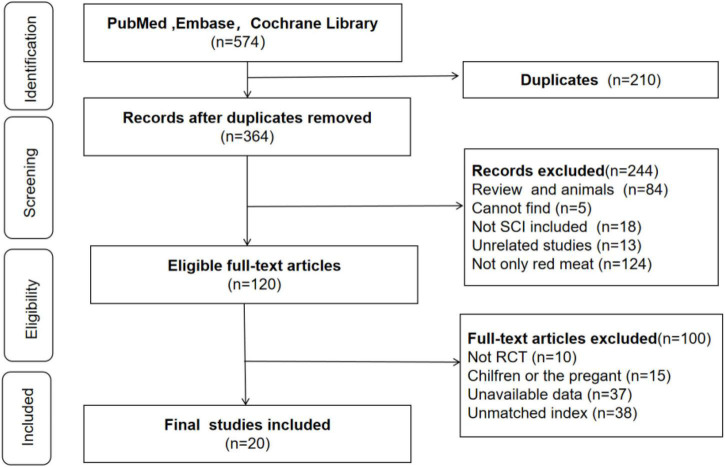
Flowchart of study selection.

We searched PubMed, EMBASE, and the Cochrane Central Register of Controlled Trials and initially found 574 studies on our research objective and first eliminated 210 duplicated studies. Then, by reading the abstracts and titles, we preliminarily excluded 244 articles. Next, we read the full text to obtain detailed information and excluded 100 articles. Finally, we included 20 studies involving 1001 people about the consumption of red meat on blood lipids (Figure 2).

### Study characteristics

The research characteristics of the 20 RCTs are presented in Tables 1–4. The studies contained relatively few participants apart from 3 studies with more than 100 participants each (29, 41, 44). The pooled data showed that all of the studies were randomized, and there were 3 studies conforming to the parallel group design (33, 41, 45). The others were crossover studies (*n* = 17). The publication years were from 1980 to 2019, with 8 articles conducted in North America, including Canada (*n* = 1) (28),USA (*n* = 3) (29, 42, 44), Houston (*n* = 1) (33), Texas (*n* = 1) (34), Quebec (*n* = 1) (40), Chicago (*n* = 1) (41), and the others were carried out in Germany (*n* = 1) (39), Iran (*n* = 2) (45, 46), Australia (*n* = 3) (31, 32, 47), and South Africa (*n* = 2) (30, 43) and Columbia (*n* = 2) (35, 36), Brazil (*n* = 1) (38). Most of the studies included both men and women (*n* = 15), except for 4 studies that included only men (28, 32–34) and 1 study only for women (37). The mean age of all participants was 22 to 59. The control group in 13 articles included white meat and in 7 articles it was legume or dairy products. The intervention duration was < 10 wk in 16 studies and ≥ 10 wk in 4 studies.

**TABLE 1:** Characteristics of the 20 RCTs.

Author	Year	Count-ry	No.of people	Gender	Healthy status	Mean Body weight (kg)	Mean BMI (kg/m^2^)	Mean age	Study Desi-gn	Control	Duration	Date Index
Beauchesne et al. (28)	2003	Canad-a	17	Men	Hypercholesterole-mia	81.4	26.5	50.1	C	Lean poultry	5wk	TC,TG, HDL-C, LDL-C
Bergeron et al. (29)	2019	USA	113	Both	Healthy, Without CAD, diabetes, other chronic disorder	NR	26	42	C	Lean white meat (8% E from chicken; 4% E from turkey	4wk	TC,TG, HDL-C, LDL-C
Wolmarans et al. (30)	1991	South Africa-n	28	Both	Healthy,BMI < 30 kg/m^2^	NR	NR	Men:35.8 Women:29.9	C	Fatty fish	6wk	HDL-C, LDL-C
Kim et al. (31)	2017	Austr-alia	49	Both	Without diabetes	NR	27	35.6	C	A diet high in whole grains, nuts, d-airy and legumes with no red meat	4wk	TC,TG,HDL-C,hs-CRP

Both: men and women; NR: not reported; R: red meat; N: non-red meat; C: crossover; P: parrallel; BMI: body mass index; wk: weeks.

**TABLE 2:** Characteristics of the 20 RCT studies (continued).

Author	Year	Country	No.of people	Gender	Healthy status	Mean Body weight (kg)	Mean BMI (kg/m^2^)	Mean age	Study Design	Control	Duration	Date Index
Asthton and Ball, (32)	2000	Australia	63	Men	Healthy, with no symptoms or prior diagnosis of CHD	NR	26.2	45.8	C	Tofu diet	4wk	TC,TG, HDL-C, LDL-C
Scott et al. (33)	1994	Houston	38	Men	Healthy, Hypercholester-olemic;	NR	NR	<50	P	Chicken	5wk	TC,TG, HDL-C, LDL-C
O’Brien and Reiser (34)	1980	Texas	29	Men	Healthy, normolipidemic	NR	NR	43	C	Fish or poultry	6wk	TC, HDL-C,
Flynn et al. (35)	1981	Columbia	38	Both	Healthy, normolipidemic	NR	NR	NR	C	Poultry	8wk	TC,TG, HDL-C
Flynn et al. (36)	1982	Columbia	21	Both	Healthy, normolipidemic	NR	R:25.5 N:25.3	R:34.0 N:36.4	C	Oily fish	12wk	TC,TG, HDL-C

Both: men and women; NR: not reported; R: red meat; N: non-red meat; C: crossover; P: parrallel; BMI: body mass index; wk: weeks.

**TABLE 3:** Characteristics of the 20 RCT studies (continued).

Author	Year	Country	No.of people	Gender	Healthy status	Mean Body weight (kg)	Mean BMI (kg/m^2^)	Mean age	Study Desig-n	Control	Duration	Date Index
Gascon et al. (37)	1996	French Canadian	14	Women	Healthy, normolipidemic	NR	22	22.4	C	Lean white fish	4wk	TC,TG, HDL-C, LDL-C
de Mello et al. (38)	2006	Brazil	17	Both	Patients with type 2 diabetes with macroalbuminuria	NR	26.2	59	C	Chicken, dairy, plant protein	4wk	TC,TG, HDL-C, LDL-C
Foerstet al. (39)	2014	German	20	Both	Healthy,without diabetes, cancer and other prevalent chronic diseases	NR	24.4	40	C	Whole grain	10wk	TC,TG, CRP
Ouellet et al. (40)	2008	Quebec	18	Both	Overweight or obese participants with insulin resistance	NR	Men:30.9 Women:33.8	Men:53.8 Women:55.4	C	Cod protein diet	8wk	TC,TG, HDL-C, LDL-C,CRP
Davidson et al. (41)	1999	Chicago	191	Both	Hypercholestero-lemia	NR	R:27.6 N:27.1	R:56.9 N:54.8	P	White meat	36wk	TC,TG, HDL-C, LDL-C

Both: men and women; NR: not reported; R: red meat; N: non-red meat; C: crossover; P: parrallel; BMI: body mass index; wk: weeks.

**TABLE 4:** Characteristics of the 20 RCT studies (continued).

Author	Year	Country	No.of people	Gender	Healthy status	Mean Body weight (kg)	Mean BMI (kg/m^2^)	Mean age	Study Desig-n	Control	Duration	Date Index
Li et al. (42)	2016	USA	34	Both	Overweight/ obese adults	R:87 N:88.1	R:31.0 N:30.7	R:51 N:56	C	Lacto-ovovegeta-rian (soy or legume)	4wk	TC,TG, HDL-C, LDL-C
Wolmarans et al. (43)	1999	South Africa	39	Both	Hyperchol-esterolemic	M:72.3 F:72.3	NR	M:35.1 F:31.5	C	Prudent diet with skinless chicken and fish	6wk	TC,TG, HDL-C, LDL-C
Hunninghake et al. (44)	2000	USA	145	Both	Hyperchol-esterolemic	NR	R:27.5 N:27.1	R:57.3 N:56.0	C	Lean white meat	36wk	TC,TG, HDL-C, LDL-C
Hassanzadeh et al. (45)	2021	Iran	44	Both	Type 2 diabetes	NR	R:26.48 N:25.69	R:56.13 N: 57.09	P	Soy bean	8wk	TC,HDL-C,LDL-C
Hosseinpour-Niazi et al. (46)	2015	Iran	31	Both	Healthy	NR	R:27.8 N:27.7	58.1	C	Legume-based TLC diet	8wk	TC,TG, HDL-C, LDL-C
Kim et al. (47)	2017	Austral-ia	51	Both	Without type2 diabetes	NR	27.7	35.1	C	A diet high in whole grains, nuts, dairy and legumes with no red meat	4wk	TC,TG, HDL-C, hs-CRP

Both: men and women; NR: not reported; R: red meat; N: non-red meat; C: crossover; P: parrallel; BMI: body mass index; wk: weeks.

### Risk of bias assessment

We conducted a quality evaluation (risk of bias) with the Cochrane risk-of-bias tool (RoB2) (Table 5). We found that all of the studies were randomized; however, only 4 studies specifically described the allocation sequence method and the allocation concealment plan. The others did not mention it. Most of the studies did not follow blinding principles, except 1 study that adopted a triple-blind design. Outcome assessors in 3 studies were not aware of the intervention assignment, and they were considered to have a low risk of bias for blinding. There were no articles with conditions such as incomplete outcomes or selective reporting, so all of the studies were considered to have a low risk of bias, and none of the studies were found to have a high risk of bias.

**TABLE 5:** Quality assessment of included studies.

Study	Random sequence generation	Allocations concealment	Blinding of participants and personnel	Blingding of outcome assessment	Incomplete outcome data	Selective outcome reporting	Other potential sources of bias	Overall
Beauchesne et al. (28)	U	U	L	L	L	L	U	U
Bergeron et al. (29)	L	L	L	L	L	L	U	L
Foerster et al. (39)	U	U	L	L	L	L	U	U
Hassanzadeh et al. (45)	L	L	L	L	L	L	U	L
Kim et al. (31)	L	L	L	L	L	L	U	L
Li et al. (42)	U	U	L	L	L	L	U	U
Wolmarans et al. (43)	U	U	L	L	L	L	U	U
Hunninghake et al. (44)	U	U	L	L	L	L	U	U
Davidson et al. (41)	U	U	L	L	L	L	U	U
Wolmarans et al. (30)	U	U	L	L	L	L	U	U
Ashton and Ball (32)	U	U	L	L	L	L	U	U
Scott et al. (33)	U	U	L	L	L	L	U	U
O’Brien and Reiser (34)	U	U	L	L	L	L	U	U
Flynn et al. (35)	U	U	L	L	L	L	U	U
Flynn et al. (36)	U	U	L	L	L	L	U	U
Gascon et al. (37)	U	U	L	L	L	L	U	U
de Mello et al. (38)	U	U	L	L	L	L	U	U
Ouellet et al. (40)	U	U	L	L	L	L	U	U
Kim et al. (47)	L	L	L	L	L	L	U	L

L: low risk of bias; H: high risk of bias; U: unclear risk of bias.

### Effects of red meat on serum lipid concentrations, inflammatory biomarkers

**FIGURE 3:**
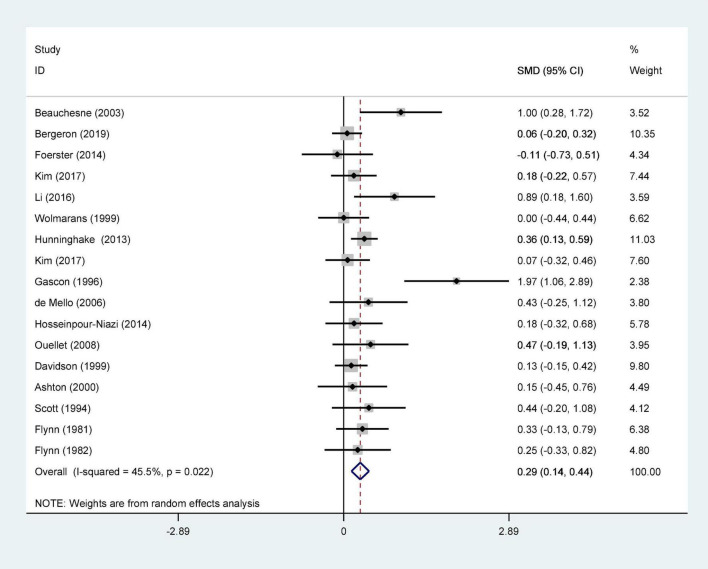
Effect of red meat consumption on TG concentration. TG, triglyceride.

**FIGURE 4:**
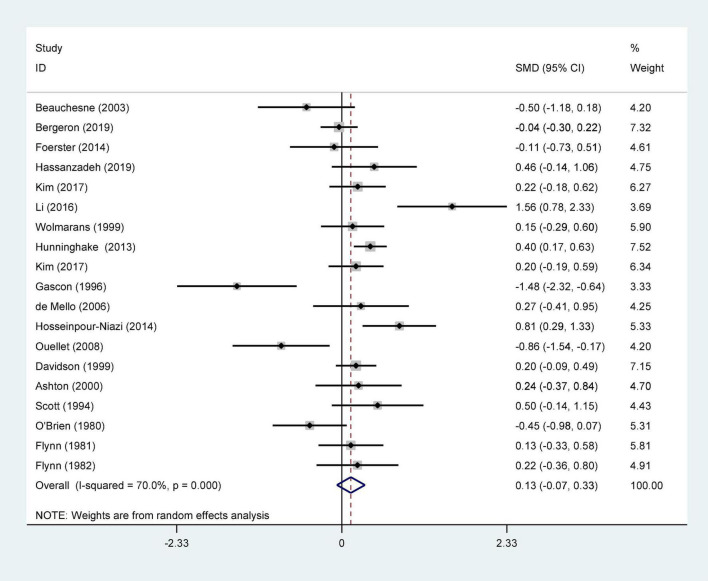
Effect of red meat consumption on TC concentration. TC, total cholesterol.

**FIGURE 5:**
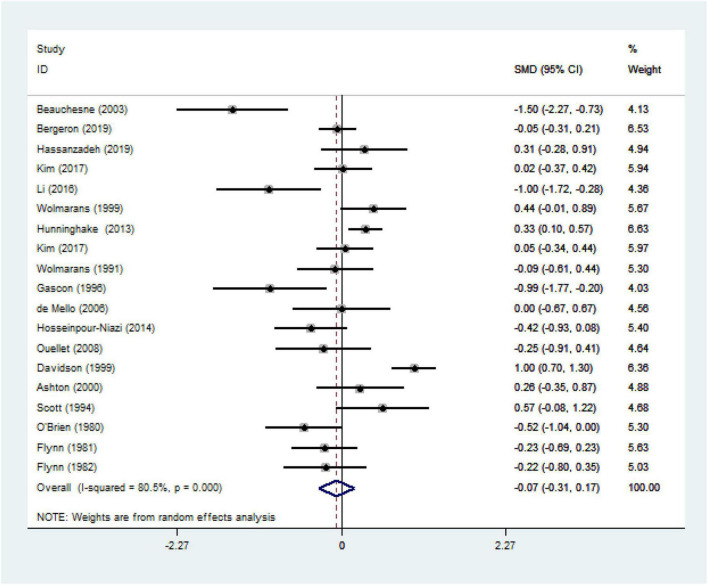
Effect of red meat consumption on HDL-C concentration. HDL-C, high-density lipoprotein cholesterol.

**FIGURE 6:**
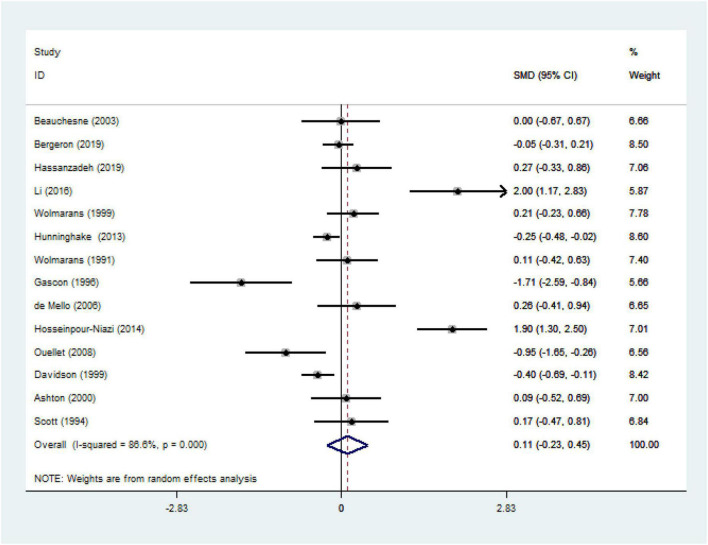
Effect of red meat consumption on LDL-C concentration. LDL-C, low-density lipoprotein cholesterol.

**FIGURE 7:**
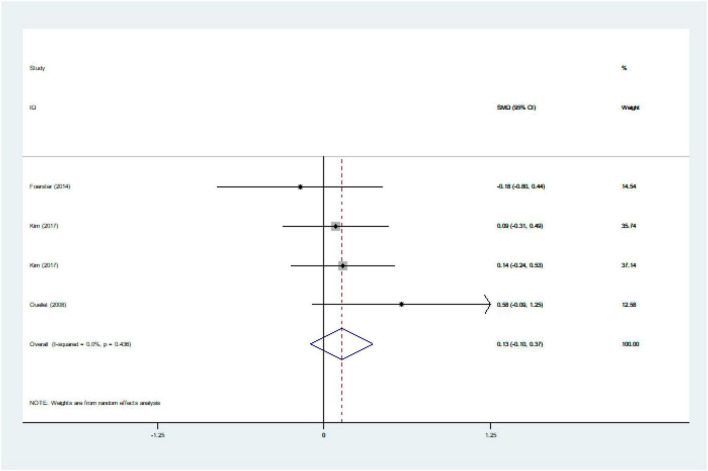
Effect of red meat consumption on CRP or hs-CRP concentration. CRP, C-reactive protein; hs-CRP, hypersensitive-CRP.

We ultimately included 17 articles on red meat consumption and serum TG levels (Figure 3), and the combined results showed that TG levels increased by approximately 0.29 mmol/L (SMD 0.29 mmol/L, 95% CI 0.14 to 0.44; *P*<0.001). The final results from 19 studies showed that red meat based diets might have no significant effects on the serum TC concentrations (SMD 0.13 mmol/L, 95% CI -0.07 to 0.33; *P* = 0.21) (Figure 4), HDL-C concentrations (SMD -0.07 mmol/L, 95% CI -0.31 to 0.17; *P* = 0.57) (Figure 5). Similarly, the overall data from 14 studies showed that red meat diets did not affect the serum LDL-C concentrations (SMD 0.11 mmol/L, 95% CI −0.23 to 0.45; *P* = 0.53) (Figure 6). The influence of red meat on the serum relative inflammatory index such as CRP or hs-CRP was reported by 4 studies, and it might be increased by approximately 0.13 mmol/L (95% CI −0.10 to 0.37; *P* = 0.273) (Figure 7), which was not statistically significant.

### Subgroup and Meta–Regression analyses

Regarding the effect of red meat on serum LDL-C, TC, TG, HDL-C, the subgroup analyses revealed that there were no reasonable subgroups to explain the moderate or high heterogeneity. We tried to explain the heterogeneity by analyzing the years, countries, number of participants, gender, BMI, age, study design, control group, and treatment period. Nevertheless, the outcome ultimately had unexplained moderate heterogeneity or relatively large differences (Tables 6, 7).

**TABLE 6:** Subgroup analyses for TC, LDL-C concentrations.

Subgroup factors	TC				LDL-C			
	No.	SMD(95% Cl)	I^2^	*P*	No.	SMD(95% Cl)	I^2^	*P*
Overall	19	0.13(-0.07,0.33)	70	0	14	0.11(-0.23,0.45)	86.6	0
**Year**								
Before 2015	14	0.03(-0.22,0.27)	70.6	0	7	-0.01(-0.40,0.37)	83.4	0
2015 or later	5	0.37(-0.02,0.76)	74.6	0.003	6	0.47(-0.07,1.00)	81.5	0
**Country**								
North America	8	0.09(-0.25,0.43)	84.7	0	5	0.45(-0.12,1.02)	88.1	0
Other	10	0.25(0.09,0.41)	0	0.657	2	0.23(-0.13,0.59)	0	0.886
**Number**								
≤ 50	14	0.09(-0.23,0.41)	75.6	0	11	0.22(-0.31,0.21)	87.2	0
¿50	5	0.19(0.02,0.36)	34.8	0.189	3	-0.23(-0.42,-0.04)	38.2	0.198
**Men**								
Men	6	0.02(-0.29,0.33)	42.5	0.122	3	0.09(-0.28,0.46)	0	0.939
Others	13	0.17(-0.08,0.42)	75.7	0	11	0.12(-0.30,0.53)	89.6	0
**BMI**								
≤ 25	2	-0.76(-2.11, 0.58)	84.8	0.01	2	-0.77(-2.55,1.02)	91.8	0
25<BMI ≤ 30	10	0.23(0.06,0.40)	43.8	0.068	8	0.18(-0.21,0.57)	86.3	0
¿30	2	0.34(-2.02,2.71)	95.2	0	2	0.51(-2.38,3.41)	96.5	0
**Age**								
¿50	9	0.13(-0.09,0.34)	54.3	0.025	6	0.15(-0.42,0.73)	90.8	0
≤ 50	8	0.11(-0.37,0.58)	83.1	0	8	0.08(-0.40,0.56)	83.4	0
**Design**								
Crossover	16	0.08(-0.16,0.31)	74	0	11	0.15(-0.29,0.58)	88.7	0
Parallel	3	0.28(0.04,0.52)	0	0.579	3	-0.05(-0.53,0.42)	63.6	0.064
**Control**								
White meat	12	-0.04(-0.28,0.20)	71.2	0	10	-0.20(-0.44,0.05)	19.5	0.293
Plant protein	6	0.43(0.11,0.76)	61.7	0.016	4	1.04(0.05,2.04)	89.5	0
**Duration**								
<10-wk	16	0.11(−0.14,0.35)	72.4	0	12	0.20(-0.24,0.64)	86.5	0
≥ 10-wk	3	0.26(0.04,0.49)	29.6	0.241	2	-0.31(-0.49,0.13)	0	0.425

No., number; SMD, standard mean difference; Cl, confidence interval; TC, total cholesterol; LDL-C, low-density lipoprotein cholesterol; BMI, body mass index.

**TABLE 7:** Subgroup analyses for TG, HDL-C concentrations.

Subgroup factors	TG				HDL-C			
	No.	SMD (95% Cl)	I^2^	*P*	No.	SMD (95% Cl)	I^2^	*P*
Overall	17	0.29 (0.14,0.44)	45.5	0.022	19	−0.07 (−0.31,0.17)	80.5	0
**Year**								
Before 2015	13	0.33 (0.14,0.53)	47	0.031	14	−0.07 (−0.40,0.25)	83.7	0
2015 or later	4	0.18 (−0.07,0.43)	37.9	0.184	5	−0.07 (−0.35,0.21)	53.3	0.073
**Country**								
North America	7	0.34 (0.13,0.56)	48	0.073	8	−0.12 (−0.59,0.35)	90.3	0
Other	9	0.15 (−0.01,0.32)	0	0.954	10	0.01 (−0.15,0.18)	9.4	0.356
**Number**								
≤ 50	12	0.40 (0.15,0.66)	54	0.013	14	−0.20 (−0.48,0.07)	68.5	0
¿50	5	0.20 (0.07,0.33)	0	0.417	5	0.23 (−0.18,0.64)	88.4	0
**Men**								
Men	5	0.39 (0.13,0.65)	0	0.447	6	−0.25 (−0.73,0.22)	75	0.001
Others	12	0.26 (0.08,0.45)	54.9	0.011	13	0.01 (-0.26,0.29)	81	0
**BMI**								
≤ 25	2	0.90 (−1.14,2.94)	92.7	0	2	−0.49 (−1.36,0.39)	71.1	0.063
25<BMI ≤ 30	9	0.21 (0.08,0.34)	9.7	0.354	10	0.05 (−0.27,0.38)	84.6	0
¿30	2	0.67 (0.18,1.15)	0	0.397	2	−0.61 (−1.35,0.12)	56.4	0.13
**Age**								
¿50	9	0.12 (−0.02,0.25)	0	0.927	8	0.12 (−0.23,0.48)	83	0
≤ 50	6	0.70 (0.28,1.12)	68	0.008	9	−0.25 (−0.67,0.18)	82.1	0
**Design**								
Crossover	15	0.31 (0.13,0.49)	50.6	0.013	16	−0.19 (−0.04,0.03)	69.4	0
Parallel	2	0.18 (−0.08,0.44)	0	0.394	3	0.69 (0.24,1.14)	57.9	0.093
**Control**								
White meat	4	0.36 (0.15,0.57)	57.7	0.009	13	−0.06 (−0.38,0.26)	84.5	0
Plant protein	5	0.17 (−0.03,0.38)	0.3	0.414	6	−0.09 (−0.42,0.23)	57.1	0.04
**Duration**								
<10-wk	14	0.34 (0.14,0.54)	51	0.014	17	−0.16 (−0.37,0.04)	62.4	0
≥ 10-wk	3	0.22 (−0.00,0.44)	29.2	0.243	2	0.66 (0.01,1.31)	91.5	0.001

No., number, SMD, standard mean difference; Cl, confidence interval; TG, triglyceride; HDL-C, high-density lipoprotein cholesterol; BMI, body mass index.

Meta-regression demonstrated that country might be a potential factor causing heterogeneity regarding to the TG levels (meta-regression *P* = 0.044). Unfortunately, meta-regression could not give a reasonable explanation of the results about the effect of red meat on the serum LDL-C, HDL-C, TC level when considering factors such as publication year, country, population size, gender, mean BMI or body weight, mean age and study design, intervention meat, control alternatives, and study duration.

### Sensitivity analysis

Sensitivity analysis indicated that the gross results of the red meat on serum lipids (TC, TG, LDL-C, HDL-C) and inflammation index (CRP or hs-CRP) were not changed by the elimination of any one study: TC (SMD changed between −0.07 and 0.33), TG (SMD changed between 0.14 and 0.44), HDL-C (SMD changed between −0.31 and 0.17), and LDL-C (SMD changed between -0.23 and 0.45), CRP (SMD changed between −0.10 and 0.37).

### Publication bias

We also evaluated publication bias through Egger’s linear regression test, and the results showed that there was no bias for TC (*P* = 0.443), LDL-C (*P* = 0.255),CRP (*P* = 0.772), but there was for TG (*P* = 0.045), or HDL-C (*P* = 0.015).

## Discussion

This meta-analysis explored the effects of red meat on serum lipid levels and inflammatory biomarkers. Our team included 20 RCTs published between 1980 and 2019. The analysis ultimately revealed that red meat consumption increased serum lipid concentrations like TG, and had no significant effects on TC, LDL-C, HDL-C, CRP, and hs-CRP.

Previous findings from a meta-analysis that included 1,803 participants in randomized controlled trials revealed that there were no significant differences among red meat, fish and low-quality carbohydrates in terms of their effects on blood lipids (48). However, it might have the potential impact on the final results because there were red meat in the comparison diets in several researches. In addition, another meta-analysis suggested that red meat, compared with non-red meat such as poultry or fish, was not necessarily correlated with increases in serum lipids; more precisely, ≥ 0.5 servings had no effect on serum lipid concentration (49). However, our research conducted subgroup analyses and the results showed that the blood lipids (TC, TG, LDL-C, HDL-C) had no direct relationship with the publication year, country, population size, gender, mean age, study design, intervention meat, control alternatives, or study duration. The only finding was that the consumption of red meat had a greater impact on the TG.

Disorders of lipid metabolism and obesity can induce higher secretion of interleukin-1β, and CRP or hs-CRP can reflect the upstream activity of inflammatory cytokines (50, 51). Meanwhile, studies have revealed that maintaining a low level of serum CRP is as important as maintaining a low serum LDL cholesterol, and statins have both anti-inflammatory and lipid-reducing functions (52–54). Elevated serum LDL cholesterol has been proven to promote the progression of coronary atherosclerotic plaques (55). They are easily oxidized under oxidative stress and turn into oxidized low-density lipoprotein (OX-LDL), which works as a damage signal in the progression of pathological conditions (56). Subsequently, macrophages release many inflammatory factors that interact with the human immune system (57–59). Overaccumulation of triglycerides in white adipose tissue will cause the release of inflammatory cytokines and has the risk of triggering systemic metabolic disease (60). In fact, medium-chain saturated fats in red meat are more likely to increase serum HDL cholesterol (16, 17). Excessive consumption of long-chain fatty acids in red meat can induce endoplasmic reticulum (ER) stress, and oxidative stress is upstream of vascular inflammation and relative dysfunction (16, 61–64).

Daily red meat consumption is often accompanied by an increased intake of NaCl, an essential nutrient for human health, which is crucial to cell homeostasis and body metabolism; however, excessive intake of NaCl can release reactive oxygen species (ROS) and have an impact on lipid metabolism, endothelial cell damage and atherosclerosis (65–67). Red meat contains more carnitine than other alternatives, and it is a metabolic precursor of trimethylamine N-oxide (TMAO), which inhibits the process of reversing cholesterol and triggers coronary artery inflammation (68–70). Carnitine is digested by the carnitine oxygenase enzyme derived from the gut microbiota into trimethylamine (TMA), which is transformed by the liver into TMAO (71). Researchers have shown that higher serum levels of TMAO after the consumption of red meat only decrease after several weeks (72).

It was proved that the nutraceuticals in daily diets could lower serum lipid levels with the help of the beneficial compounds (73). Carotenoids and resveratrol, which mainly exist in the fruits, vegetables diets and Mediterranean foods, are able to work as anti-inflammatory molecules in the management of lipid disorders to prevent cardiovascular diseases (74, 75). Proanthocyanidins are also proved to reduce the triacylglycerol concentration in the blood (76). Similarly, Water-insoluble fish proteins (IFP) is beneficial for dyslipidaemia treatment through lowering serum cholesterol (77). Fish oil are demonstrated to be rich in unsaturated fatty acids which are good for reducing triacylglycerol levels (78).

## Strengths and limitations

Our research not only extracted data on serum lipids but also paid attention to the relative inflammatory index. Inflammation is a potential risk factor for various chronic diseases and related basic causes (8–10). This review collected relevant inflammatory indicators to explore the potential impact of inflammation on blood lipids. In addition, all of the articles included in this study were RCTs with a high level of evidence. Moreover, our research performed subgroup analyses and meta-regression to verify the potential link between possible factors and blood lipids regarding the consumption of red meat. The outcome of the meta-regression indicated that country might be a potential factor to give rise to heterogeneity with regard to TG levels. Regarding the various diet habits in the different areas and differences among studies, we are supposed to further analyze the heterogeneity and be cautious about this outcome. Sensitivity analysis indicated that the gross results did not change with the elimination of any one study. Publication bias was assessed through Egger’s linear regression test. Considering that there were not enough relevant articles were included, we consider that the publication bias is related to the number of articles, and we advise caution about the results. This review could provide a useful reference for clinical treatment and disease prevention

However, our study had the following limitations. Notably, there was no deny that there was a higher heterogeneity involved in our study and we applied a random-effects model for statistical analyses, subgroup analyses and meta-regression were adopted to explain the heterogeneity. Meta-regression revealed that different countries might be the potential factors to induce the heterogeneity regarding to the TG levels. However, there were no reasonable subgroups to explain the moderate or high heterogeneity for serum lipids (TC, TG, LDL-C, HDL-C) and Egger’s linear regression test also showed the publication bias for TGs and HDL-C. Undeniably, the limited articles included might be the potential risk factors. Meanwhile, further large-scale researches should be explored in the future and we might be cautious about the results.

In addition, eating habits and lifestyle are crucial to health (4, 5, 79). We lacked data about the quantity of red meat and the proportion of energy obtained from protein and ignored daily habits. Moreover, due to different personal habits and hobbies, the studies could not be double-blinded, possibly causing bias. Different countries and regions had different ways of cooking food; these different ways and cooking oils might have potential effects on lipids, and we could not analyse these effects nor could we analyze different food additives (5, 80, 81). Therefore, future studies should include various processing methods and additives. A larger sample size is also necessary.

## Conclusion

In conclusion, the pooled results of our meta-analysis showed that the consumption of red meat might increase the serum lipid concentrations, especially for TG concentration,. but had a little affect on TC, LDL-C, HDL-C and CRP or hs-CRP Therefore, considering the effect of red meat on blood lipids, we hold a negative opinion about eating red meat, especially for people with a higher TG concentration. In addition, future studies will advocate larger number of participants, clarify the quantities, cooking methods, in order to ensure the safety of red meat on lipid profiles.

## Data availability statement

The original contributions presented in this study are included in the article/supplementary material, further inquiries can be directed to the corresponding author/s.

## Author contributions

LS first proposed the suggestion under the guidance of C-XH. J-LY and W-KX were responsible for conducting the search, screening articles, and extracting the data. J-HL and G-PM assessed the quality of the articles. LS, X-KC, SW, and X-XZ performed the statistical analysis. LS wrote the article. Q-CC and HW were responsible for the final revision. C-XH was the guarantor of the entire content. All authors reviewed and agreed with the content of this article.
